# Genome-Wide cfDNA Methylation Profiling Reveals Robust Hypermethylation Signatures in Ovarian Cancer

**DOI:** 10.3390/cancers17122026

**Published:** 2025-06-17

**Authors:** Simone Karlsson Terp, Karen Guldbrandsen, Malene Pontoppidan Stoico, Lasse Ringsted Mark, Anna Poulsgaard Frandsen, Karen Dybkær, Inge Søkilde Pedersen

**Affiliations:** 1 Department of Molecular Diagnostics, Aalborg University Hospital, 9000 Aalborg, Denmark; 2 Department of Clinical Medicine, Aalborg University, 9000 Aalborg, Denmark; 3 Clinical Cancer Research Center, Aalborg University Hospital, 9000 Aalborg, Denmark; 4 Department of Obstetrics and Gynecology, Aalborg University Hospital, 9000 Aalborg, Denmark; 5 Department of Pathology, Aalborg University Hospital, 9000 Aalborg, Denmark; 6 Department of Hematology, Aalborg University Hospital, 9000 Aalborg, Denmark

**Keywords:** DNA methylation, Cell-free DNA, biomarkers, ovarian cancer, epigenetics

## Abstract

**Background:** Ovarian cancer remains the most lethal gynecological cancer, primarily due to its asymptomatic nature in early stages and consequent late diagnosis. Early detection improves survival, but current biomarkers lack sensitivity and specificity. Cell-free DNA (cfDNA) released from tumor cells captures tumor-associated epigenetic alterations and represents a promising source for minimally invasive biomarkers. Among these, aberrant DNA methylation occurs early in tumorigenesis and may reflect underlying disease biology. This study aimed to investigate genome-wide cfDNA methylation profiles in patients with ovarian cancer, benign ovarian conditions, and healthy controls to identify cancer-associated methylation patterns that may inform future biomarker development. **Results:** We performed genome-wide cfDNA methylation profiling using cell-free methylated DNA immunoprecipitation sequencing (cfMeDIP-seq) on plasma samples from 40 patients with high-grade serous ovarian carcinoma, 38 patients with benign ovarian conditions, and 38 healthy postmenopausal women. A total of 536 differentially methylated regions (DMRs) were identified between ovarian cancer and controls (n = 76), with 97% showing hypermethylation in ovarian cancer. DMRs were enriched in CpG islands and gene bodies and depleted in repetitive elements, consistent with known cancer-associated methylation patterns. Fifteen genes showed robust hypermethylation across analyses. These genes exhibited methylation across intronic, exonic, and upstream regulatory regions. Separate comparisons of ovarian cancer to each control group (benign and healthy) supported the reproducibility of these findings. Gene Ontology enrichment analysis revealed enrichment in gland development, embryonic morphogenesis, and endocrine regulation, suggesting biological relevance to ovarian tumorigenesis. **Conclusions:** This study identifies consistent cfDNA hypermethylation patterns in ovarian cancer, affecting genes involved in developmental regulation and hormone-related processes. Our findings underscore the potential of cfMeDIP-seq for detecting tumor-specific methylation signatures in plasma and highlight these 15 hypermethylated genes as biologically relevant targets for future studies on cfDNA methylation in ovarian cancer.

## Simple Summary

Ovarian cancer is often diagnosed at an advanced stage due to a lack of early symptoms and insufficient screening tools. Earlier detection could improve outcomes, but current blood-based biomarkers are not sensitive or specific enough. In this study, we analyzed cell-free DNA from blood samples to investigate whether DNA methylation patterns could distinguish ovarian cancer from benign ovarian conditions and healthy individuals. DNA methylation is a chemical modification that often changes in cancer and can reflect underlying disease processes. Using a genome-wide approach, we identified regions with increased methylation in ovarian cancer, many of which were located in genes involved in development and hormone regulation. These patterns were consistent across analyses and revealed distinct methylation profiles between cancer and non-cancer samples. Our findings support further research into DNA methylation alterations as a source of biomarker candidates and offer insight into the biological changes associated with ovarian cancer.

## 1. Introduction

Ovarian cancer (OC) is the most lethal gynecological malignancy, with only modest improvements in survival rates over the last two decades [1,2]. This is primarily due to its asymptomatic nature in the early stages, leading to most cases being diagnosed at an advanced stage, where complete surgical resection is rarely achievable [3,4]. Consequently, the prognosis remains poor, with an overall five-year survival rate of 49% [2]. In contrast, when detected early, the five-year survival rate is above 90% [5]. Despite extensive research, reliable biomarkers for early detection are still lacking. Cancer antigen 125 (CA125) and transvaginal ultrasound have been widely explored as potential biomarkers, but their accuracy remains insufficient and has not translated into improved overall survival [6,7]. This underscores the urgent need for more reliable biomarkers to detect OC at an earlier stage when treatment is more likely to be effective.

Cell-free DNA (cfDNA) released from tumor cells, known as circulating tumor DNA (ctDNA), has emerged as a promising biomarker for the early detection of cancer, offering real-time insights into the genetic and epigenetic landscape of the tumor [8,9]. Among epigenetic alterations, aberrant DNA methylation at cytosine–phosphate–guanine (CpG) sites is an early and frequent hallmark of tumorigenesis, influencing gene regulation and genome stability [10,11]. Many cancers show a characteristic pattern of global hypomethylation in repetitive and intergenic regions, alongside localized hypermethylation of CpG islands in gene promoters, which can lead to the silencing of tumor suppressor genes [12]. These widespread and functionally important alterations make DNA methylation a compelling target for biomarker discovery. In OC, several cfDNA methylation biomarkers have been investigated [9], but none has yet been implemented in clinical practice for early diagnosis. Although genome-wide methylation analyses of cfDNA have been performed for OC [13,14], these studies primarily focused on developing diagnostic classification algorithms to distinguish cancer from controls, with limited exploration of the underlying biological mechanisms. Further studies are, therefore, needed to comprehensively characterize tumor-associated methylation patterns and investigate the biological relevance of epigenetic alterations that can distinguish OC from non-cancer controls.

One promising approach for detecting low-abundance methylation changes in cfDNA is cell-free methylated DNA immunoprecipitation sequencing (cfMeDIP-seq) [15]. This method has previously identified methylation changes in various cancer types, including ovarian, breast, renal, and gastric cancer [13,16,17,18], and has been proposed as a promising method for detecting methylation alterations in plasma and may support further non-invasive biomarker discovery.

In this study, we utilized cfMeDIP-seq to characterize genome-wide cfDNA methylation profiles in OC, benign ovarian conditions, and healthy controls to uncover cancer-associated epigenetic alterations that may inform future efforts to distinguish OC from non-cancer controls.

## 2. Methods

### 2.1. Patient Cohort and Sample Collection

Blood samples were obtained from 40 OC patients with histologically verified high-grade serous carcinoma (HGSC) at the time of diagnosis, prior to any surgery or treatment, at Aalborg University Hospital. Only patients with primary OC and no history of other malignancies were included. Samples were collected in EDTA tubes between 2011 and 2020. For comparative analysis, blood samples were also collected from 40 patients with benign ovarian conditions and 40 healthy female blood donors. The benign cohort comprised patients who underwent surgery due to high clinical suspicion of OC but were subsequently diagnosed with histologically verified benign ovarian conditions with no prior or concurrent cancers. The selection was designed to reflect the proportional distribution of benign diagnoses typically observed among women undergoing surgery for suspected OC, thereby enhancing the clinical relevance of the comparator group. EDTA blood samples from the benign cohort were obtained between 2009 and 2020, at the time of diagnosis, before any surgical or treatment intervention, also at Aalborg University Hospital. Healthy donor EDTA blood samples were collected in 2025 from the Aalborg University Hospital Blood Bank and included only postmenopausal females aged 50 years or older. Due to failed library preparation resulting in insufficient sequencing reads, two samples from each of the benign and healthy control groups were excluded, leaving 38 samples per group for downstream analyses. Detailed participant characteristics of the included individuals are provided in Table 1.

Plasma was isolated within two hours of blood draw by centrifugation at 2000× *g* for 10 min at 18 °C using a slow brake and stored at −80 °C until further use. The study was approved by the Danish Health Research Ethics Committee (NVK-2114546).

### 2.2. cfDNA Extraction

cfDNA was extracted from 1 to 2.1 mL of plasma (mean: 1.6 mL, SD: ±0.36 mL) using the QIAamp Circulating Nucleic Acid Kit (Qiagen, Hilden, Germany) on the QIAcube platform (Qiagen), following the manufacturer’s protocol. The cfDNA was eluted in 40 µL Buffer AVE (Qiagen) and stored at −20 °C. Quantification was carried out using a Qubit 1X dsDNA HS Assay Kit (ThermoFisher Scientific, Waltham, MA, USA) on a Qubit Flex Fluorometer (ThermoFisher Scientific), yielding concentrations between 0.04 and 3.73 ng/µL (mean: 0.40 ng/µL, SD: ±0.45 ng/µL).

### 2.3. cfDNA Quality Control

Quality assessment of cfDNA samples was performed using Droplet Digital PCR (ddPCR, Bio-Rad, Hercules, CA, USA) with a ddPCR Multiplex cfDNA Quality Control Assay, following the protocol described in Terp et al. [19]. Briefly, each 22 µL ddPCR reaction contained 1× ddPCR Multiplex Supermix (Bio-Rad), primers and probes [20,21,22] (sequences listed in Table S1), and 2 µL of template DNA input. Droplets were generated using an Automated Droplet Generator (Bio-Rad), and PCR was performed on a C1000 Touch Thermal Cycler (Bio-Rad) with the following thermal cycling conditions: initial denaturation at 95 °C for 10 min; 40 cycles of 94 °C for 30 s and 55 °C for 1 min; and final enzyme deactivation at 98 °C for 10 min, with a ramp rate of 1 °C/s throughout all steps. The PCR plate was further incubated at 4 °C overnight before droplet reading. After droplet reading on the QX600 Droplet Reader (Bio-Rad), absolute quantification (copies/µL) was determined using QX Manager Software v2.2 (Bio-Rad). All samples were analyzed in technical duplicates, and positive and negative controls were included in each ddPCR run in duplicates. Only wells with a minimum of 10,000 accepted droplets were included for analysis to ensure data quality.

Contamination with high molecular weight (HMW) DNA was evaluated by calculating the percentage of lymphocyte-derived DNA (PBC assay) relative to total cfDNA (RPP30 assay) using the formula (PBC copies/RPP30 copies) × 100. A sample with <0.5% contamination was considered acceptable, whereas a sample with >2% would be flagged as contaminated [19]. Additionally, cfDNA integrity was assessed by calculating the ratio of long to short amplicon copies, with ratios <0.4 considered acceptable and >0.7 indicative of contamination [19,22].

### 2.4. cfMeDIP-Seq

The cfMeDIP-seq libraries were prepared following a modified version of the protocol described by Shen et al. [23]. To avoid a batch effect, both OC, benign, and healthy samples were included in each cfMeDIP-seq library run. Input DNA amounts ranged from 1.0 to 93.3 ng (mean: 9.9 ng, SD: ±11.2 ng). Library preparation was performed using 0.5× volumes of KAPA HyperPrep Kit reagents (Roche, Basel, Switzerland) in combination with xGen UDI-UMI Adapters (IDT, Coralville, IA, USA). Following adapter ligation, an immunoprecipitation step was performed to selectively enrich for methylated cfDNA using a 5-methylcytosine antibody (Clone 33D3, Diagenode, Liège, Belgium). The enriched methylated cfDNA underwent purification with the IPure Kit V2 (Diagenode) to remove contaminants and unbound DNA fragments. Library amplification was conducted with KAPA HiFi Hotstart ReadyMix (Roche) and Illumina P5 and P7 primers (IDT, primer sequences available in Table S2), with subsequent clean-up with 1.0× AMPure XP beads (Beckman Coulter, Brea, CA, USA). The final libraries were eluted in 44 µL of nuclease-free water, and the quality and quantification were assessed using the TapeStation HS kit (Agilent Technologies, Santa Clara, CA, USA). Libraries were sequenced on an S2 flow cell using an Illumina NovaSeq 6000 system (San Diego, CA, USA) with 150 bp paired-end reads. Unique molecular identifiers (UMIs) were incorporated during library preparation and used for deduplication.

### 2.5. cfMeDIP-Seq Data Processing

Illumina NovaSeq 6000 output files were converted from BCL to FASTQ formats using BCL Convert version 4.0.3 (Illumina), with index barcodes being identified and marked in the process. The resulting FASTQ reads were processed using MEDIPIPE, a pipeline designed for cfMeDIP-seq processing by Zeng et al. [24]. Sequencing reads were adapter-trimmed with Trim Galore (version 0.6.7), followed by alignment to the human reference genome (version hg38) using BWA (version 0.7.17). Duplicate reads were removed, and BAM files were indexed using SAMtools (version 1.17). As part of the pipeline, methylation levels were estimated by dividing the reads into 300 bp non-overlapping bins using MEDIPS (version 1.52.0) [25]. Data quality was assessed with MEDIPS [25] by evaluating CpG coverage, CpG enrichment, and coverage saturation.

## 3. Data Analysis

### 3.1. Filtering Process

Initial filtering steps were applied to ensure high-quality methylation data. Bins overlapping genomic regions from the ENCODE Blacklist (ENCFF356LFX) [26], known to contain problematic and artifact-prone regions, were excluded [27]. 

To reduce background methylation noise, methylation signals derived from peripheral blood leukocytes (PBLs) of non-cancer controls (n = 20) from Hag et al. [28] were used to filter the cfDNA methylation profiles. As PBLs are a major source of cfDNA in circulation, regions with high methylation signal in PBLs were considered likely to reflect hematopoietic background rather than ctDNA [28]. Specifically, bins with more than 20 read counts in a non-cancer PBL sample were identified and excluded from all cfDNA samples prior to downstream analyses.

Additionally, bins with total read counts below 10 across the whole dataset were removed to reduce noise from low-coverage regions. Bins without CpG sites were also excluded to ensure that only high-confidence methylation signals were examined.

### 3.2. Singular Value Decomposition Analysis

To assess potential batch effects related to library preparation, singular value decomposition (SVD) analysis was performed on the filtered cfDNA methylation data. Variance-stabilizing transformation (VST) was applied prior to decomposition using the DESeq2 R package (version 1.44.0). The resulting principal components were evaluated for association with library preparation batches, and a one-way analysis of variance (ANOVA) was performed to determine whether technical variation contributed significantly to the observed data structure [29,30].

### 3.3. K-Means Clustering Analysis

Unsupervised k-means clustering was performed to investigate whether cfDNA methylation profiles could distinguish OC samples by disease stage. Specifically, the analysis evaluated whether FIGO stage II and stage III samples formed distinct clusters, reflecting stage-associated differences in methylation patterns. Methylation count data from OC samples were filtered to include the 10,000 most variable bins based on row-wise variance. K-means clustering (k = 2) was applied to the transposed count matrix to assign samples to two clusters. Principal component analysis was then performed to visualize the clustering, with FIGO stage annotations overlaid to assess the relationship between cluster assignment and disease stage.

### 3.4. Differentially Methylated Region Analysis

Differentially methylated region (DMR) analysis between OC and controls (comprising both healthy and benign samples) was performed using the DESeq2 R package (version 1.44.0). The Wald test was applied to assess differential methylation between groups, and *p*-values were adjusted for multiple testing using the Benjamini–Hochberg method to control the false discovery rate. The control group was set as the reference level in the model. Bins with an adjusted *p*-value (padj) < 0.1 were considered statistically significant. A relaxed significance threshold (padj < 0.1) was used to account for the expected sparsity of the cfDNA methylation signal and to capture biologically meaningful changes in an exploratory genome-wide context. A volcano plot was generated to visualize the distribution of hypo- and hypermethylated regions.

### 3.5. Annotation of DMRs

DMRs were annotated using the annotatr R package (version 1.30.0) to identify overlaps with genomic features. CpG feature annotations included CpG islands, shores, shelves, and open sea regions based on the hg38 reference genome. Gene features such as promoters, exons, introns, untranslated regions (UTRs), and intergenic regions were annotated using annotatr with the hg38_basicgenes track. Repetitive elements were annotated with UCSC RepeatMasker (version 2022-10-18) and classified into long interspersed nuclear elements (LINEs), short interspersed nuclear elements (SINEs), long terminal repeat elements (LTRs), which included retroposons, and other repeat types. In addition, DMRs were assigned to genes based on genomic overlap using the EnsDb.Hsapiens.v86 annotation database (version 2.99.0) and the GenomicRanges package (version 1.56.2).

### 3.6. Permutation Analysis and Genomic Feature Enrichment

To evaluate whether the overlap between hypermethylated DMRs and specific genomic features was greater than expected by chance, permutation analysis was performed using the randomizeRegions function from the regioneR package (version 1.36.0). This function randomly redistributed the observed hypermethylated DMRs across the genome while preserving their length and chromosomal distribution. For each permutation (n = 1000), overlaps between randomized regions and genomic annotations were recalculated.

Separate analyses were performed for CpG features (islands, shores, shelves, and open sea), repeat elements (LINEs, SINEs, LTRs, and other repeats), and gene features (promoters, exons, introns, intergenic regions, and UTRs). For each feature, the number of overlaps in the real dataset was compared to the null distribution of counts from the 1000 permuted sets. Z-scores were calculated to quantify the deviation of the observed overlap from the random expectation. Positive Z-scores indicate enrichment and negative Z-scores indicate depletion relative to the null distribution. Z-scores above 2 or below −2 were interpreted as strong evidence of enrichment or depletion, respectively.

### 3.7. Heatmap

To illustrate methylation differences between OC and control groups, a heatmap was generated based on the 15 robustly hypermethylated DMRs, each mapped to a unique gene. For genes with multiple associated DMRs, the DMR with the lowest adjusted *p*-value was selected to represent that gene. Methylation count data for the selected regions were extracted from the filtered dataset and log2-transformed. The resulting matrix was row-scaled and visualized using the pheatmap R package (version 1.0.12). To maintain the clinical grouping structure, sample columns were ordered by diagnostic group rather than clustered.

### 3.8. Pathway and Gene Ontology Enrichment Analysis

Functional enrichment analysis was conducted using the clusterProfiler R package (version 4.12.6). Genes associated with hypermethylated DMRs (log_2_ fold change > 0, adjusted *p*-value < 0.1) were converted to Entrez gene IDs prior to enrichment testing. For the Kyoto Encyclopedia of Genes and Genomes (KEGG) and Gene Ontology (GO) enrichment analyses, terms with an adjusted *p*-value and q-value below 0.1 were considered significantly enriched. *p*-values were corrected for multiple testing using the Benjamini–Hochberg method.

### 3.9. Statistical Analysis

Group differences in clinical variables were assessed using appropriate statistical tests. Age differences among the three groups (OC, benign, and healthy) were analyzed using one-way ANOVA. Body mass index (BMI), which did not follow a normal distribution, was compared across groups using the Kruskal–Wallis test. For pairwise comparisons between OC and benign, the Wilcoxon rank-sum test was used to assess differences in CA125 levels and the Risk of Malignancy Index (RMI). Differences in cfDNA input amounts for cfMeDIP-seq between groups were also assessed using the Kruskal–Wallis test.

All statistical analyses and data visualizations were performed in R (version 4.4.3).

## 4. Results

In this study, the cfDNA methylation profiles of 40 patients with OC were examined using peripheral blood samples collected at diagnosis, prior to surgery and treatment. Of these patients, 30% (n = 12) were diagnosed with stage II disease and 70% (n = 28) with stage III disease (Table 1). In addition, cfDNA methylation profiles were examined for two age-matched control groups: 38 patients with benign ovarian conditions and 38 healthy postmenopausal females. The benign group consisted of individuals who underwent surgery due to suspected OC but were ultimately diagnosed with benign ovarian conditions, providing a clinically relevant comparison group. The age and BMI of patients with OC did not differ significantly from those of the benign and healthy control groups (age: ANOVA, *p* = 0.22; BMI: Kruskal–Wallis, *p* = 0.38). In contrast, CA125 and RMI levels were significantly elevated in patients with OC compared to those with benign conditions (CA125: Wilcoxon rank-sum test, *p* < 0.0001; RMI: Wilcoxon rank-sum test, *p* < 0.0001). Detailed participant characteristics are summarized in Table 1.

For all samples with sufficient material, quality control parameters for high-quality cfDNA were fulfilled. Specifically, no evidence of HMW DNA contamination was observed, as assessed using a ddPCR Multiplex Quality Control assay (Table S3). One sample could not be assessed due to insufficient available material. The cfDNA input for cfMeDIP-seq was comparable between groups (Kruskal–Wallis test, *p* = 0.062). Following this, cfDNA methylation profiling was performed using cfMeDIP-seq. All samples met internal quality control criteria for high-quality cfMeDIP-seq data and were included in downstream analyses (Table S3). To assess potential batch effects related to library preparation runs, we examined the association between library preparation and the top principal components of variation identified through SVD. No significant batch effects were observed (SV1: ANOVA, *p* = 0.732; SV2: ANOVA, *p* = 0.401), and samples were evenly distributed across library preparation runs in the SVD plot (Figure S1).

To explore whether the cfDNA methylation profiles differed between stage II and stage III OC cases, unsupervised k-means clustering and principal component analysis were performed based on the top 10,000 most variable methylated bins. Clustering initially revealed a distinct sample (OC40), which formed its own cluster and corresponded to a stage III case (Figure S2). To assess whether this sample influenced stage-specific clustering, the analysis was repeated after excluding OC40. The rerun clustering showed no distinct separation between stages (Figure S3), suggesting that stage-specific methylation patterns were not dominant in this dataset. Therefore, all OC samples, including OC40, were analyzed as a single group in subsequent differential methylation analyses.

**Figure 1:**
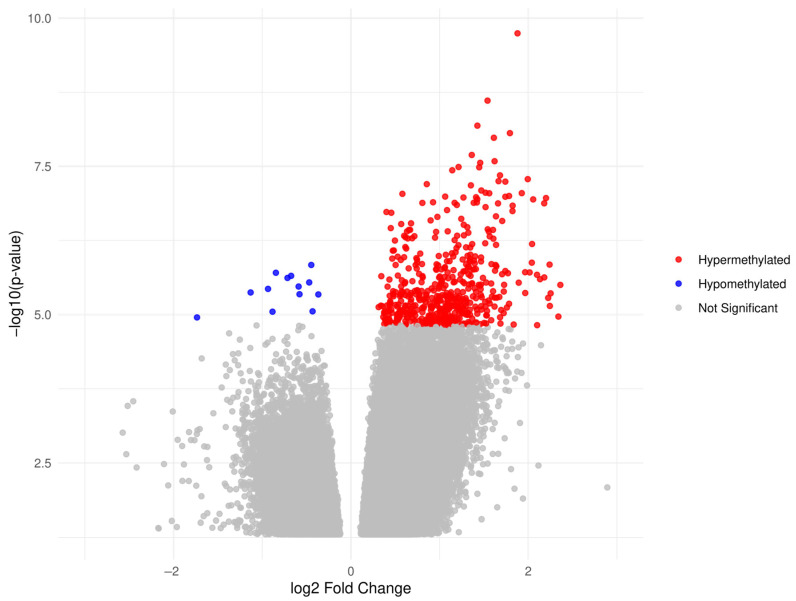
Volcano plot of DMRs between OC samples (n = 40) and controls (benign and healthy samples, n = 76). Each point represents a genomic region (bins of 300 bp size), assessed by cfMeDIP-seq. The x-axis shows the log2 fold change in methylation between groups, and the y-axis shows the −log10 (*p*-value). Regions with a false discovery rate (padj) below 0.1 are highlighted. Hypermethylated regions in OC are shown in red, hypomethylated regions in blue, and non-significant regions in gray. bp: base pair, cfMeDIP-seq: cell-free methylated DNA immunoprecipitation sequencing, DMRs: differentially methylated regions, OC: ovarian cancer.

To compare cfDNA methylation profiles between OC and control groups, we performed a DMR analysis using DESeq2 (Figure 1). For this analysis, the benign and healthy control groups were combined into a single control group (n = 76) to increase statistical power and represent the broader non-cancer cfDNA methylation landscape. The analysis identified a total of 536 significant DMRs (padj < 0.1), of which 523 were hypermethylated and 12 were hypomethylated in OC. Notably, one OC sample (OC40) exhibited a consistently high methylation signal across many DMRs and contributed substantially to the total number of significant regions identified. Further investigation revealed that OC40 had been sequenced at a substantially deeper depth than other samples due to a flow cell loading error rather than reflecting intrinsic biological differences such as tumor burden or CA125 levels. To assess the robustness of the differential methylation results, the DMR analysis was repeated with OC40 excluded. This analysis identified 30 DMRs that remained significant at padj < 0.1, annotating 17 genes. Of these 17 genes, 15 overlapped with the gene set identified in the original analysis, including all OC cases. These 15 genes were, therefore, considered robust markers of OC-associated methylation changes. Based on these findings, OC40 was retained in the analyses to capture the full biological spectrum of cfDNA methylation patterns in OC.

**Figure 2:**
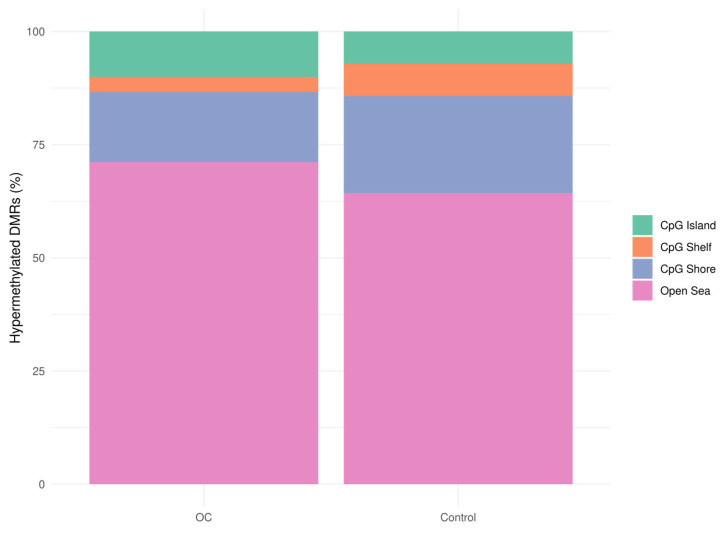
Bar plot of the distribution of hypermethylated DMRs (padj < 0.1) across the CpG features, CpG islands (green), CpG shores (blue), CpG shelves (orange), and open sea regions (pink), in OC (n = 40) and control samples (benign and healthy samples, n = 76). DMRs: differentially methylated regions, OC: ovarian cancer.

To characterize the distribution of hypermethylated regions across CpG features, we examined their overlap with CpG islands, CpG shores, CpG shelves, and open sea regions. Compared to controls, hypermethylated DMRs in OC were more frequently located in CpG islands (OC: 10% vs. controls: 7.1%) and open sea regions (OC: 71.1% vs. controls: 64.3%), with fewer regions found in CpG shores (OC: 15.5% vs. controls: 21.4%) and CpG shelves (OC: 3.4% vs. controls: 7.1%) (Figure 2). Permutation analysis further revealed significant enrichment of hypermethylated DMRs in CpG islands (Z = 22.2) and shores (Z = 15.1), significant depletion in open sea regions (Z = −14.3), and no enrichment in CpG shelves (Z = 0.3) in OC samples (Figure S4), supporting the tumor-specific methylation patterns detected by cfMeDIP-seq.

**Figure 3:**
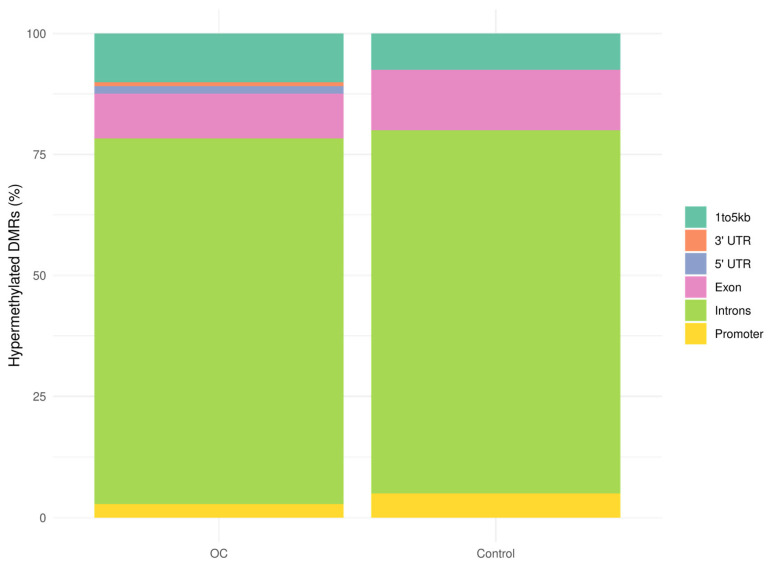
Bar plot of the distribution of hypermethylated DMRs (padj < 0.1) overlapping various gene features in OC samples (n = 40) and control samples (benign and healthy samples, n = 76). 1–5 kb: 1 to 5 kb from transcription start site, DMRs: differentially methylated regions, OC: ovarian cancer, UTR: untranslated region.

Next, we examined the distribution of hypermethylated DMRs across gene features. The majority of hypermethylated DMRs in both OC and control samples were located in intronic regions (OC: 75.5% vs. controls: 75%). Smaller proportions were found in exons (OC: 9.3% vs. controls: 12.5%), promoters (OC: 2.8% vs. controls: 5%), and regions 1–5 kb upstream of transcription start sites (OC: 10.1% vs. controls: 7.5%) (Figure 3). Notably, hypermethylated DMRs in the 3′ and 5′ UTRs were observed only in the OC group (3′UTRs: 0.8%, 5′UTRs: 1.5%). Permutation analysis revealed significant enrichment of hypermethylated DMRs in intronic regions (Z = 5.0), 5′ UTRs (Z = 4.1), exons (Z = 3.3), and 1–5 kb upstream regions (Z = 3.5) in OC, while DMRs in promoters were modestly enriched (Z = 1.8), and 3′ UTRs showed no enrichment (Z = −0.2) (Figure S5). These findings suggest a widespread distribution of tumor-associated methylation changes across regulatory and noncoding gene regions.

**Figure 4:**
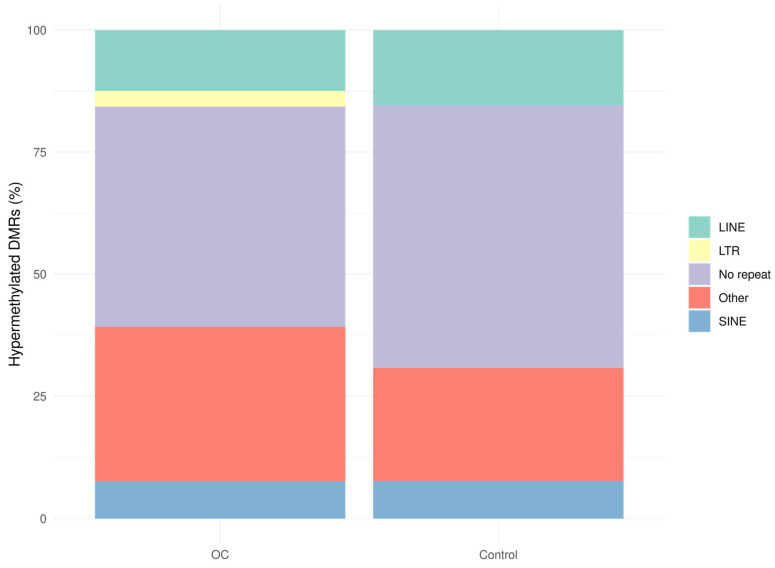
Bar plot of the distribution of hypermethylated DMRs (padj < 0.1) across repetitive elements in OC samples (n = 40) and control samples (benign and healthy samples, n = 76). DMRs: differentially methylated regions, LINE: long interspersed nuclear element, LTR: long terminal repeat element, OC: ovarian cancer, SINE: short interspersed nuclear element.

We also examined the distribution of hypermethylated DMRs across repetitive elements to assess whether tumor-associated methylation changes were enriched in specific repeat features. The majority of DMRs in OC were located in regions without repetitive elements (45.1%), similar to controls (53.8%) (Figure 4). Among annotated repeats, the “other repeat” category was most frequently represented in both groups (OC: 31.5% vs. controls: 23.1%), followed by LINEs (OC: 12.4% vs. controls: 15.4%) and SINEs (OC: 7.7% vs. controls: 7.7%). Notably, hypermethylated DMRs in LTRs were only observed in OC samples (3.3%), suggesting a possible tumor-specific methylation signal in these elements. Permutation analysis showed significant depletion of hypermethylated DMRs in LINEs (Z = −4.0), SINEs (Z = −5.3), and other repeat elements (Z = −3.0), with slight enrichment observed in LTRs (Z = 0.8) and no deviation from the expected distribution in non-repetitive regions in OC samples (Figure S6). These results are consistent with global hypomethylation of repetitive sequences observed in cancer [12] and further support the tumor specificity of cfMeDIP-seq-derived methylation profiles.

To explore the biological relevance of the methylation changes, we annotated each DMR to overlapping genes based on genomic coordinates using the Ensemble-based gene annotation database EnsDb.Hsapiens.v86. This resulted in a total of 274 unique genes annotated to significant hypermethylated DMRs (padj < 0.1) (Table S4).

As described above, a reanalysis excluding the OC40 sample confirmed the robustness of key findings, with 15 genes consistently identified in both analyses. We, therefore, focused on these 15 robustly hypermethylated genes (Table 2). Several of the robust DMRs showed substantial methylation differences between OC and controls, with log₂ fold changes ranging from 0.9 to 1.9 (Table 2), corresponding to approximately 1.9- to 3.7-fold increases in methylation signal. The majority of DMRs associated with these 15 genes were located in intronic regions (Table S5), with additional DMRs overlapping exonic regions, promoters, UTRs, and 1–5 kb upstream regions (Table S5). This distribution suggests that tumor-associated methylation changes occur across both coding and regulatory gene features in OC.

**Figure 5:**
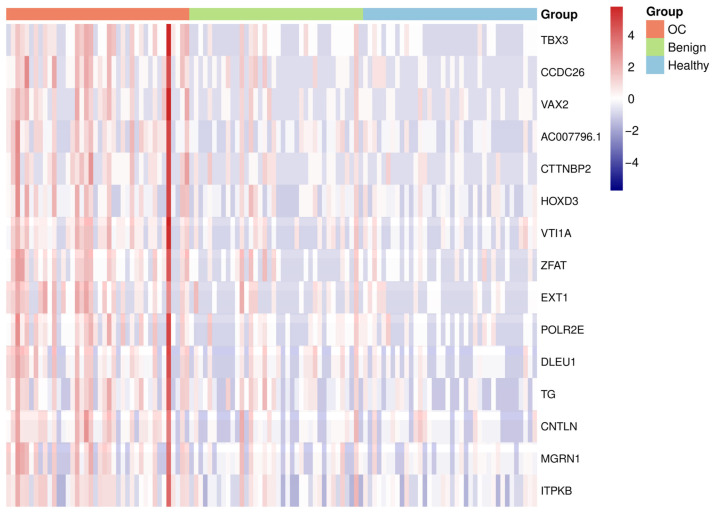
Heatmap showing log₂-transformed methylation signal of 15 robustly hypermethylated gene-annotated DMRs across all samples. Color intensity reflects the scaled signal per gene (row), and samples are grouped by diagnosis (column) without column clustering. OC samples are shown in red, benign in green, and healthy controls in blue. DMRs: differentially methylated regions, OC: ovarian cancer.

The differential methylation patterns observed in the 15 robustly hypermethylated genes are illustrated in a heatmap showing their methylation signal across all samples (Figure 5). Genes are ordered according to Table 2, and samples are manually grouped by diagnosis to facilitate comparison between groups. Although the sample groups were manually ordered, the overall signal patterns revealed clear group-level differences, with OC samples exhibiting markedly higher methylation compared to controls. Healthy controls showed consistently low methylation signals, while benign samples displayed greater variability, with some individuals presenting intermediate methylation patterns between healthy and OC profiles. These patterns indicate that hypermethylation at these genes is primarily associated with malignant transformation. One OC sample (OC40) displayed consistently elevated methylation across all 15 genes, consistent with the pattern identified in the unsupervised clustering analysis (Figure S2), likely attributable to the higher sequencing depth of OC40 rather than underlying biological heterogeneity.

To further assess the consistency of the observed methylation differences, we conducted separate DMR analyses comparing OC samples to either benign (n = 38) or healthy controls (n = 38) alone. As expected, these subgroup comparisons identified fewer significant DMRs due to reduced sample sizes. In the OC vs. benign comparison, fifteen DMRs were identified (thirteen hypermethylated, two hypomethylated, Figure S7), including *MTA3*, *SCN8A*, *CASC11*, and *IQSEC3*, which were also detected in the OC vs. combined controls analysis (Table S4). *SV2C* was unique to the OC vs. benign comparison. In the OC vs. healthy comparison, thirteen DMRs were identified (eleven hypermethylated, two hypomethylated, Figure S8), including *TG*, *HOXD3*, *CCDC26*, and *AC007796.1*, which were among the fifteen robustly hypermethylated genes (Table S4). Additional overlaps included *SLC12A7* and *GOLGA8B*, while *ACOXL* was unique to this comparison. Despite the reduced number of significant regions in these subgroup analyses, the presence of several overlapping genes, including members of the robust gene set, reinforces the reproducibility and biological relevance of key OC-associated methylation changes.

**Figure 6:**
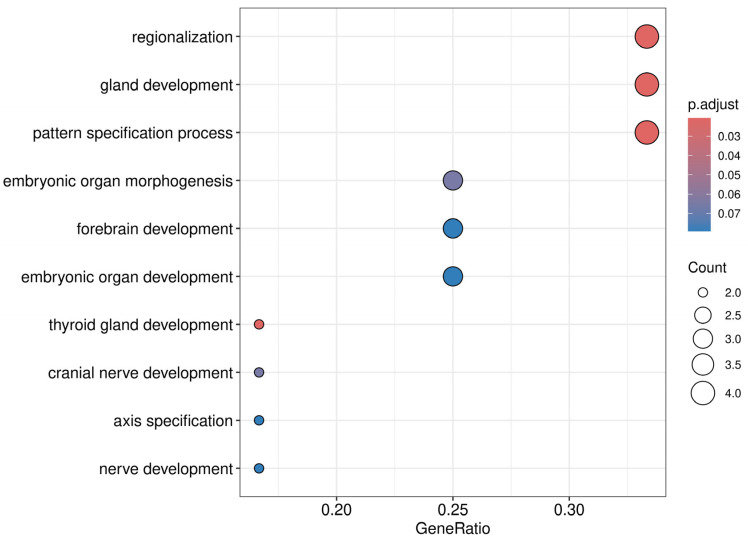
Dot plot showing the top 10 significantly enriched GO biological processes based on the 15 robustly hypermethylated genes in OC. The x-axis represents the GeneRatio, calculated as the number of hypermethylated genes involved in a given GO term divided by the total number of genes tested. The dot size reflects the number of genes contributing to each term (count), and the color indicates the adjusted *p*-value (p.adjust), with more significant values shown in red. GO: Gene Ontology, OC: ovarian cancer.

To explore the biological relevance of the 15 robustly hypermethylated genes, we performed GO biological process enrichment analysis. The analysis revealed several significantly enriched terms (padj < 0.1), including gland development, thyroid gland development, regionalization, embryonic organ morphogenesis, and pattern specification process (Figure 6, Table S6). These findings suggest that the observed methylation changes may be linked to pathways involved in developmental and endocrine processes, which are increasingly recognized as relevant to ovarian tumor biology [31,32,33]. KEGG pathway analysis was also performed on the 15 robustly hypermethylated genes but did not yield any significantly enriched pathways, likely due to the small gene set size, which limits the sensitivity of pathway-level analysis in KEGG.

## 5. Discussion

In this study, we profiled genome-wide cfDNA methylation patterns in patients with OC, benign ovarian conditions, and healthy postmenopausal controls using cfMeDIP-seq. We identified 536 DMRs between OC and controls, the vast majority (97%) of which were hypermethylated. These hypermethylated regions were predominantly located in CpG islands and gene bodies and depleted from repetitive elements, consistent with known cancer-associated epigenetic alterations [12,34]. Notably, OC samples displayed consistent and distinct hypermethylation patterns compared to controls. Importantly, the identification of 15 robustly hypermethylated genes remained consistent even after excluding OC40, which had unusually high sequencing depth, reinforcing the reproducibility of these findings. Supporting this, separate subgroup analyses comparing OC to either benign or healthy controls alone also identified DMRs. Several of the 15 robustly methylated genes, such as *TG*, *HOXD3*, *CCDC26*, and *AC007796.1*, were also detected in the OC vs. healthy comparison. In addition, several other genes that were significantly methylated in the OC vs. combined control analysis also overlapped with the OC vs. benign and OC vs. healthy comparisons. Although the number of significant DMRs was reduced due to smaller sample sizes, the presence of overlapping genes across these independent comparisons supports the broader reproducibility of the identified methylation changes. These results suggest the presence of distinct and reproducible cfDNA methylation signatures associated with malignant transformation in OC.

To better understand the functional implications of the identified hypermethylated regions, we reviewed existing evidence related to the 15 genes consistently hypermethylated in cfDNA from OC patients. For most genes in our study, hypermethylation was concentrated within gene bodies, including exons, introns, and UTRs, rather than promoter regions. Gene body methylation has been associated with active transcription, alternative splicing, and transcript stability in cancer and normal tissues [34,35,36]. This broader distribution of methylation suggests that the epigenetic dysregulation observed in OC may influence gene expression through multiple mechanisms beyond transcriptional silencing. Future studies could explore the functional consequences of these methylation changes by analyzing matched tumors and benign ovarian tissues using RNA sequencing or quantitative PCR. Several genes identified have previously been implicated in cancer development and progression. *TBX3*, a transcriptional repressor that inhibits the p53 pathway by repressing ARF and CIP1 [37], has been reported to be hypermethylated in bladder cancer, where its methylation is associated with a worse prognosis [38]. *TBX3* also suppresses E-cadherin expression, enabling epithelial tumor cell invasion [37]. Consistent with the broader methylation pattern observed, *TBX3* hypermethylation in our study was detected in exonic and 3′ UTRs, suggesting a potential role in post-transcriptional regulation. *CCDC26*, a long noncoding RNA, regulates DNA methylation by controlling DNMT1 localization [39]. Disruptions in *CCDC26* have been linked to leukemia and pancreatic cancer [40,41], with elevated expression associated with poor prognosis [41]. In our study, *CCDC26* was predominantly hypermethylated within intronic regions, supporting a role for gene body methylation in cancer-related gene regulation. *VAX2* is a transcription factor linked to cancer progression and dysregulation. Promoter hypermethylation of *VAX2* has been associated with reduced gene expression and higher tumor grade in bladder cancer [42]. In contrast, in gastric cancer, *VAX2* is upregulated and promotes proliferation and metastasis by repressing tumor suppressor genes [43]. In our study, *VAX2* hypermethylation was found both 1 to 5 kb upstream of the transcription start site and within intronic regions, suggesting that methylation of both distal regulatory elements and gene bodies may contribute to its dysregulation in OC. While *AC007796.1* remains poorly characterized, its inclusion in an OC risk signature [44] supports its potential functional relevance. *CTTNBP2* was hypermethylated across upstream, exonic, intronic, and promoter regions in our study. In OC, *CTTNBP2* upregulation promotes proliferation, migration, and invasion and inhibits senescence [45]. It has also been implicated in tumor progression in glioma, prostate, and bladder cancers [46,47,48], suggesting a broader oncogenic role. The homeobox gene *HOXD3* was hypermethylated across 5′ UTR, exonic, intronic, and promoter regions. Hypermethylation of *HOXD3* has been associated with reduced expression and poor prognosis in OC [49] and renal cancer [50] and has demonstrated diagnostic potential using cfDNA in prostate cancer [51].

Beyond these more extensively characterized genes, additional hypermethylated genes were identified. *VTI1A* is implicated in cancer risk through fusion events, particularly in glioma [52], although its role in methylation-driven regulation remains unclear; *ZFAT* is involved in apoptosis and angiogenesis and is detected in hematologic cancer cell lines [53], exhibiting copy number alterations in OC [54]; and *EXT1* is predominantly hypermethylated within intronic regions in our study and is associated with WNT signaling regulation and tumor progression in lung and uterine cancers [55,56]. *ITPKB*, involved in calcium signaling and reactive oxygen species regulation, has also been observed methylated in nasopharyngeal carcinoma [57]. *POLR2E*, encoding an RNA polymerase II subunit, has been associated with worse survival outcomes [58], while *DLEU1*, an lncRNA, is highly expressed in OC and promotes proliferation and invasion [59]. *TG* (thyroglobulin) hypermethylation has been associated with aggressive tumor behavior in breast cancer [60], and *CNTLN*, hypermethylated within gene bodies in our study, has been detected in epithelial OC cell lines [61], although its functional role remains unclear. 

Notably, *MGRN1* emerged as particularly relevant to OC. Hypermethylation of the upstream region of *MGRN1* has been linked to platinum resistance and poor survival in HGSC, likely through the dysregulation of EGR1-mediated pathways [62]. Consistent with these findings, our study observed hypermethylation of *MGRN1* in promoter regions and 1 to 5 kb upstream to the transcription start site and more broadly across exonic and intronic regions. 

The hypermethylation patterns observed across these genes suggest widespread epigenetic dysregulation in OC. These findings provide biological insight into tumor development and identify biologically relevant regions that warrant further investigation in the context of cfDNA-based biomarker research.

To explore the functional relevance of the 15 robustly hypermethylated genes, we performed a GO enrichment analysis focused on biological processes. The analysis identified several significantly enriched terms (padj < 0.1), including gland development, thyroid gland development, embryonic organ morphogenesis and development, and regionalization, among others. Notably, these findings align with the growing recognition that tumorigenesis and embryonic development share substantial biological parallels, including overlapping gene expression programs, epigenetic regulation, and signaling pathways [63]. This overlap supports the concept that tumor cells may reactivate developmental programs to promote malignancy [63]. Likewise, the enrichment of endocrine-related processes is consistent with the established role of hormonal regulation in ovarian carcinogenesis and prognosis [64,65,66,67]. 

This broader functional context supports the notion that epigenetic dysregulation in OC extends beyond canonical oncogenic pathways and may involve the dysregulation of developmental and endocrine programs critical for tissue specification and homeostasis. In contrast, KEGG pathway enrichment analysis did not yield significant results for the same gene set. This is likely due to the small number of genes analyzed (n = 15), which limits the statistical power of pathway-based methods, such as KEGG. These findings highlight the utility of GO enrichment in uncovering relevant biological themes from smaller, focused gene sets.

To our knowledge, no previous cfMeDIP-seq studies in OC have specifically examined the biological relevance of hypermethylated genes identified through genome-wide profiling. The only published cfMeDIP-seq study in OC primarily focused on classification model development without in-depth analysis of the underlying biological processes [13]. By contrast, our study provides the first comprehensive functional annotation of consistently hypermethylated regions in OC, offering insights into potential epigenetic mechanisms of tumor development and progression and contributing a biological foundation for future investigations into cfDNA methylation in this context.

This study has several strengths. We performed genome-wide methylation profiling using cfMeDIP-seq, allowing an unbiased assessment of epigenetic alterations across the cfDNA landscape. The cases consisted only of patients with HGSC. The terminology OC covers a heterogeneous group of cancers, and including all would make interpretation of the results difficult. Our inclusion of two control groups, healthy postmenopausal women and patients with benign ovarian conditions who were initially suspected of having malignancy, enhances the clinical relevance of our comparisons and strengthens the potential diagnostic relevance of the findings. In addition, we accounted for background signals from normal hematopoietic cells by subtracting PBL methylation profiles, which helped reduce noise and enhance tumor specificity. Furthermore, all sample groups were included in each library preparation run to minimize technical bias. Batch effects were formally assessed, and none were detected. Finally, since DNA methylation is known to change with age [68], we confirmed that age distributions were similar between groups, reducing the likelihood of age-related confounding.

Despite these strengths, several limitations should be acknowledged. While we accounted for PBL-derived methylation signal using a public dataset, we did not include matched peripheral blood samples from the same patients. Although prior work has shown that using patient-matched PBLs can improve tumor specificity in cfDNA methylation analyses [28], such samples were not collected at the time of diagnosis in our cohort. For future prospective cohort studies, however, including patient-matched PBL samples at the time of blood collection would enable more precise background correction and enhance tumor-specific signal detection. Our study included only patients with stage II and III OC, limiting conclusions about early-stage disease detection. In addition, the modest sample size and exploratory nature of the study, which was not designed to assess clinical performance metrics, such as sensitivity and specificity, limit the generalizability of our findings and highlight the need for validation in larger, independent cohorts. Although the cohort size allowed us to detect reproducible methylation differences between groups, it may limit statistical power for identifying subtler effects, particularly in subgroup analyses. As such, our findings should be considered hypothesis generating. Moreover, as we did not include cfDNA from patients with other cancer types, we cannot determine whether the observed methylation changes are specific to OC or reflect more general cancer-associated patterns.

Future studies are needed to validate the 15 robustly hypermethylated genes identified in this study in larger, independent cohorts of OC patients, ideally including early-stage OC cases, to determine their consistency and potential biological relevance across disease stages. The inclusion of matched PBL samples in such cohorts would further improve background correction and tumor specificity in cfDNA methylation analyses. Moreover, combining selected hypermethylated regions into a weighted methylation score could be explored in future studies to evaluate their ability to distinguish OC from non-cancer controls. While this would require statistical modeling and independent validation, it represents a promising direction for cfDNA-based biomarker development.

Taken together, our findings provide new insights into the epigenetic landscape of OC as reflected in cfDNA and highlight the potential of genome-wide methylation profiling to reveal tumor-associated methylation changes associated with OC in plasma cfDNA.

## 6. Conclusions

In this study, we utilized cfMeDIP-seq to characterize genome-wide cfDNA methylation profiles in OC, benign ovarian conditions, and healthy controls, aiming to uncover cancer-associated epigenetic alterations that may distinguish malignant from non-malignant cases. We identified distinct and predominantly hypermethylated regions in OC, including 15 robustly hypermethylated genes. These findings provide new insights into the epigenetic landscape of OC as reflected in cfDNA and support the potential of genome-wide methylation profiling to inform future biomarker research.

## Figures and Tables

**Table 1:** Participant characteristics for the study. Categorical variables are reported as counts with percentages in parentheses. BRCA1/2 mutation status was obtained from prior analyses of tumor tissue. BMI: body mass index, CA125: cancer antigen 125, FIGO: International Federation of Gynecology and Obstetrics, NA: not available, OC: ovarian cancer, RMI: Risk of Malignancy Index.

	OC Cohort (n = 40)	Benign Cohort (n = 38)	Healthy Cohort (n = 38)
**Age**, median [range]	67.5 [48–85]	62.5 [19–91]	62 [53–73]
**BMI**, median [range]	24.3 [18.1–48.2]	25.9 [13.1–35.1]	25.7 [19.6–35]
**Postmenopausal**	37 (92.5%)	29 (76.3%)	38 (100%)
**FIGO 2013 stage at diagnosis**		NA	NA
II	12 (30%)		
III	28 (70%)		
**Histology, malignant**		NA	NA
High-grade serous carcinoma	40 (100%)		
**Histology, benign**	NA		NA
Serous cystadenoma/adenofibroma		17 (44.7%)	
Mucinous cystadenoma/adenofibroma		5 (13.2%)	
Benign Brenner tumor		2 (5.3%)	
Cyst not otherwise specified		1 (2.6%)	
Mature teratoma/struma ovarii		3 (7.9%)	
Endometriosis		4 (10.5%)	
Fibroma/thecoma		3 (7.9%)	
Inflammation/abscess		2 (5.3%)	
Other reactive changes		1 (2.6%)	
**RMI**, median [range]	2164 [75–75,474]	231 [48–3807]	NA
**RMI ≥ 200**	37 (92.5%)	22 (57.9%)	NA
**CA125 (kU/L)**, median [range]	391 [20–8386]	50 [13–513]	NA
**Tobacco use**		NA	
Current	6 (15%)		3 (7.9%)
Previous	12 (30%)		14 (36.8%)
Never	21 (52.5%)		21 (55.3%)
Unknown	1 (2.5%)		0 (0%)
**Parity**		NA	
0	4 (10%)		4 (10.5%)
1	4 (10%)		5 (13.2%)
≥2	32 (80%)		29 (76.3%)
**BRCA1/2 mutation**		NA	NA
Yes	12 (30%)		
No	27 (67.5%)		
Unknown	1 (2.5%)		

**Table 2:** Robustly hypermethylated DMRs in OC annotated to genes. Adjusted *p*-values were calculated using the Benjamini–Hochberg method to account for multiple comparisons. For genes with multiple associated DMRs, the DMR with the lowest adjusted *p*-value was selected to represent that gene. The 15 genes shown were consistently hypermethylated in the full dataset and after excluding the OC40 sample, indicating their robustness across analyses. DMRs: differentially methylated regions. Chr: chromosome. Log_2_FC: log_2_ fold change.

Gene Name	Chr	Start Site	Stop Site	Log_2_FC	Adjusted *p*-Value
TBX3	Chr 12	114,671,701	114,672,000	1.9	0.0006
CCDC26	Chr 8	129,582,001	129,582,300	1.8	0.0074
VAX2	Chr 2	70,907,401	70,907,700	1.9	0.0128
AC007796.1	Chr 19	31,352,701	31,353,000	1.5	0.0044
CTTNBP2	Chr 7	117,792,601	117,792,900	1.7	0.0128
HOXD3	Chr 2	176,163,601	176,163,900	1.7	0.0128
VTI1A	Chr 10	112,815,601	112,815,900	1.6	0.0116
ZFAT	Chr 8	134,687,701	134,688,000	1.5	0.0116
EXT1	Chr 8	117,931,801	117,932,100	1.4	0.0116
POLR2E	Chr 19	1,087,801	1,088,100	1.5	0.0128
DLEU1	Chr 13	50,133,001	50,133,300	1.4	0.0116
TG	Chr 8	132,882,901	132,883,200	1.3	0.0208
CNTLN	Chr 9	17,444,101	17,444,400	1.1	0.0128
MGRN1	Chr 16	4,664,101	4,664,400	1.1	0.0150
ITPKB	Chr 1	226,680,901	226,681,200	0.9	0.0128

## Data Availability

The sequencing data generated and analyzed during the current study are not publicly available, as they contain information that could compromise the privacy of the research participants. However, they are available from the corresponding author upon reasonable request and following review and approval by the Danish Health Research Ethics Committee.

## Supplementary Materials

The following supporting information can be downloaded at https://www.mdpi.com/article/10.3390/cancers17122026/s1, Figure S1. Plot of singular value decomposition analysis to assess potential batch effects. Figure S2. Plot of K-means clustering with all OC samples. Figure S3. Plot of K-means clustering without OC40. Figure S4. Plot of Z scores for CpG features. Figure S5. Plot of Z scores for Gene features. Figure S6. Plot of Z scores for Repeat features. Figure S7. Volcano plot of DMRs between OC and benign. Figure S8. Volcano plot of DMRs between OC and healthy. Table S1. Table with primer and probe sequences for the ddPCR Multiple cfDNA QC assay. Table S2. Table with primer sequences for Illumina P5 and P7 primers. Table S3. Excel file with ddPCR Multiplex cfDNA QC results and QC results from cfMeDIP-seq. Table S4. Excel file with all DMRs. Table S5. Gene features for 15 robustly hypermethylated genes. Table S6. Excel file with Gene Ontology processes.

## Abbreviations

ANOVA	Analysis of variance
PBLs	Peripheral blood leukocytes
BMI	Body mass index
bp	Base pair
CA125	Cancer antigen 125
cfDNA	Cell-free DNA
cfMeDIP-seq	Cell-free methylated DNA immunoprecipitation sequencing
CpG	Cytosine–phosphate–guanine
ctDNA	Circulating tumor DNA
ddPCR	Droplet digital PCR
DMR	Differentially methylated region
FIGO	Federation of Gynecology and Obstetrics
GO	Gene ontology
HGSC	High-grade serous carcinoma
HMW	High molecular weight
KEGG	Kyoto Encyclopedia of Genes and Genomes
LINEs	Long interspersed nuclear elements
LTRs	Long terminal repeat elements
NA	Not applicable
OC	Ovarian cancer
RMI	Risk of malignancy index
SINEs	Short interspersed nuclear elements
SVD	Singular value decomposition
UMIs	Unique molecular identifiers
UTR	Untranslated region
VST	Variance-stabilizing transformation
