# Supplementary material for: Genome-Wide cfDNA Methylation Profiling Reveals Robust Hypermethylation Signatures in Ovarian Cancer
Source: Cancers (Basel). 2025 Jun 17;17(12):2026. doi: 10.3390/cancers17122026 (PMC12190857; doi:10.3390/cancers17122026)
Supplement: Supplementary file 1 [file cancers-17-02026-s001.zip › Table S1 - Table with primer and proble sequences for the ddPCR Multiple cfDNA QC assay.pdf]

Table S1 - Table with Primer and probe sequences for the ddPCR Multiplex cfDNA QC assay.

Assay	Assay component	Sequence (5'-3')	Amplicon length (bp)	Ref
RPP30	Forward primer	GATTTGGACCTGCGAGCG	62	(19)
	Reverse primer	GCGGCTGTCTCCACAAGT		
	Probe	HEX-TTCTGACCTGAAGGCTCTGCG		
PBC	Forward primer	ATCTGCAAATGAACAGYCTGAGA	90-125	(20)
	Reverse primer	CTTACCTGAGGAGACGGTGAC		
	Probe	FAM-CYGAGGACACRGCTGTGTATTACTGTGC		
EMC7 250	Forward primer	AAGTACTACTGAGTATGATGTT	250	(21)
	Reverse primer	CTAGATTTGCCAGATGATTTT		
	Probe	Cy5-AGTTGCCTGATGTTTCTGAGTTCAT		
EMC7 65	Forward primer	CTTTCCCCATGTTGCTTTAT	65	(21)
	Reverse primer	CTGACAACCTCTGATGTTTT		
	Probe	Cy5.5-CAGAGCAAGATATGTGAATTACATCAA		
