# Supplementary material for: Genome-Wide cfDNA Methylation Profiling Reveals Robust Hypermethylation Signatures in Ovarian Cancer
Source: Cancers (Basel). 2025 Jun 17;17(12):2026. doi: 10.3390/cancers17122026 (PMC12190857; doi:10.3390/cancers17122026)
Supplement: Supplementary file 1 [file cancers-17-02026-s001.zip › Table S2 - Table with primer sequences for Illumina P5 and P7 primers.pdf]

Table S2 - Table with primer sequences for Illumina P5 and P7 primers.

**Primer**	**Sequence**
P5	AAT GAT ACG GCG ACC ACC GAG AT
P7	CAA GCA GAA GAC GGC ATA CGA
