# Supplementary material for: Genome-Wide cfDNA Methylation Profiling Reveals Robust Hypermethylation Signatures in Ovarian Cancer
Source: Cancers (Basel). 2025 Jun 17;17(12):2026. doi: 10.3390/cancers17122026 (PMC12190857; doi:10.3390/cancers17122026)
Supplement: Supplementary file 1 [file cancers-17-02026-s001.zip › Table S5 - Gene features for 15 robustly hypermethylated genes.pdf]

Table S5 - Gene features annotation of hypermethylated DMRs associated with the 15 robustly hypermethylated genes. DMRs: differentially methylated regions, Number of DMRs: number of DMRs annotated to the gene within a specific gene feature, NA: not available, 1 to 5 kb upstream: 1 to 5 kb upstream of transcription start site, UTR: untranslated region.

**Gene name**	**Gene feature**	**Number of DMRs**
CCDC26	Intron	15
CNTLN	Exon	1
CNTLN	Intron	3
CTTNBP2	1 to 5 kb upstream	1
CTTNBP2	Exon	9
CTTNBP2	Intron	5
CTTNBP2	Promoter	2
DLEU1	Intron	20
EXT1	Intron	4
HOXD3	5' UTR	2
HOXD3	Exon	2
HOXD3	Intron	12
HOXD3	Promoter	2
ITPKB	Intron	4
MGRN1	1 to 5 kb upstream	5
MGRN1	Exon	12
MGRN1	Intron	39
MGRN1	Promoter	3
POLR2E	3' UTR	3
POLR2E	Exon	3
POLR2E	Intron	2
TBX3	3' UTR	2

TBX3	Exon	2
TG	Exon	7
TG	Intron	21
VAX2	1 to 5 kb upstream	1
VAX2	Intron	2
VTI1A	3' UTR	2
VTI1A	Exon	2
ZFAT	Intron	30
AC007796.1	No feature annotated	NA
