# Supplementary figures and images for: Genome-Wide cfDNA Methylation Profiling Reveals Robust Hypermethylation Signatures in Ovarian Cancer
Source: Cancers (Basel). 2025 Jun 17;17(12):2026. doi: 10.3390/cancers17122026 (PMC12190857; doi:10.3390/cancers17122026)

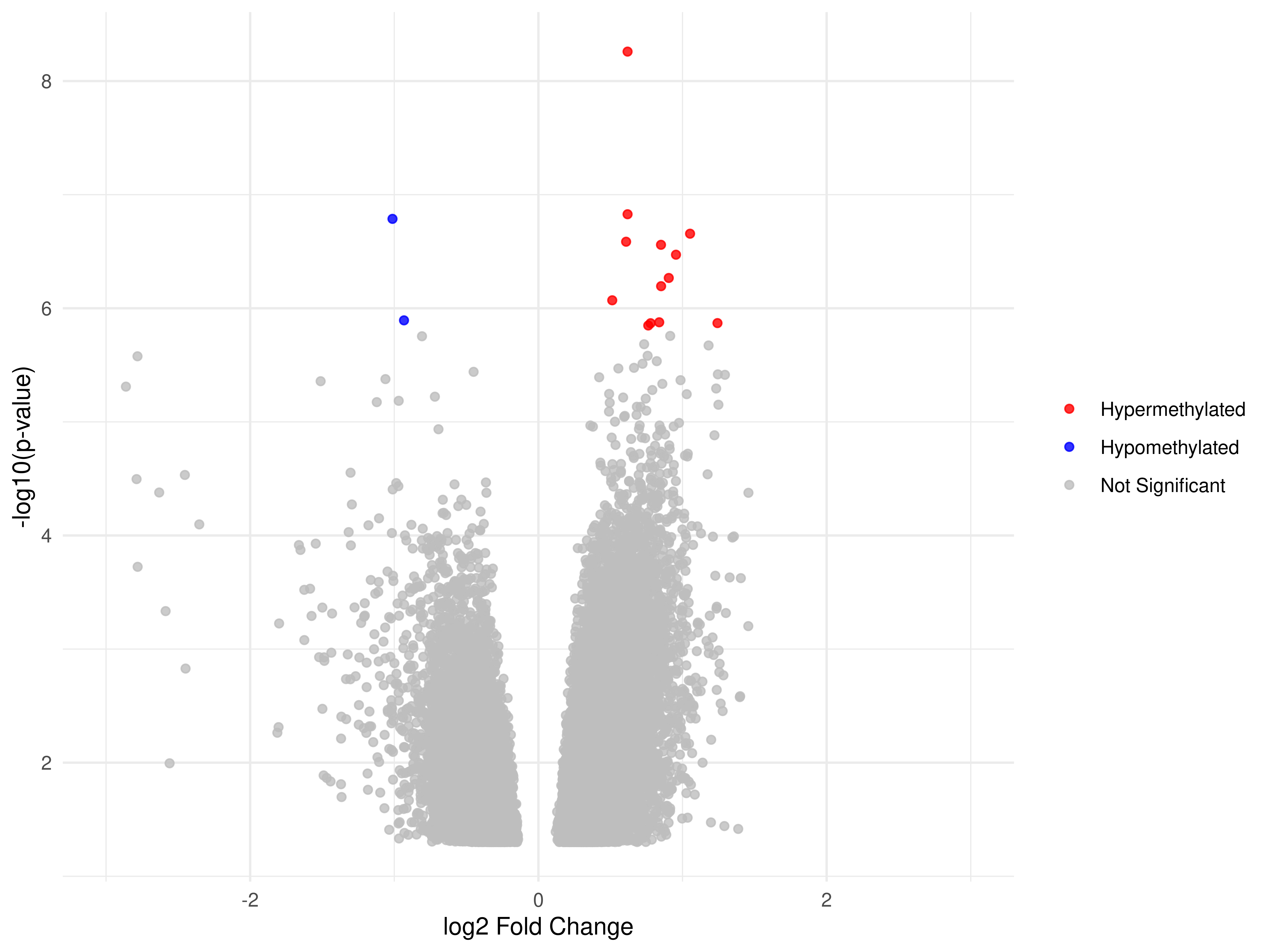

Supplement: Supplementary file 1 [file cancers-17-02026-s001.zip › Figure S7 - Volcano plot of DMRs between OC and benign.png]

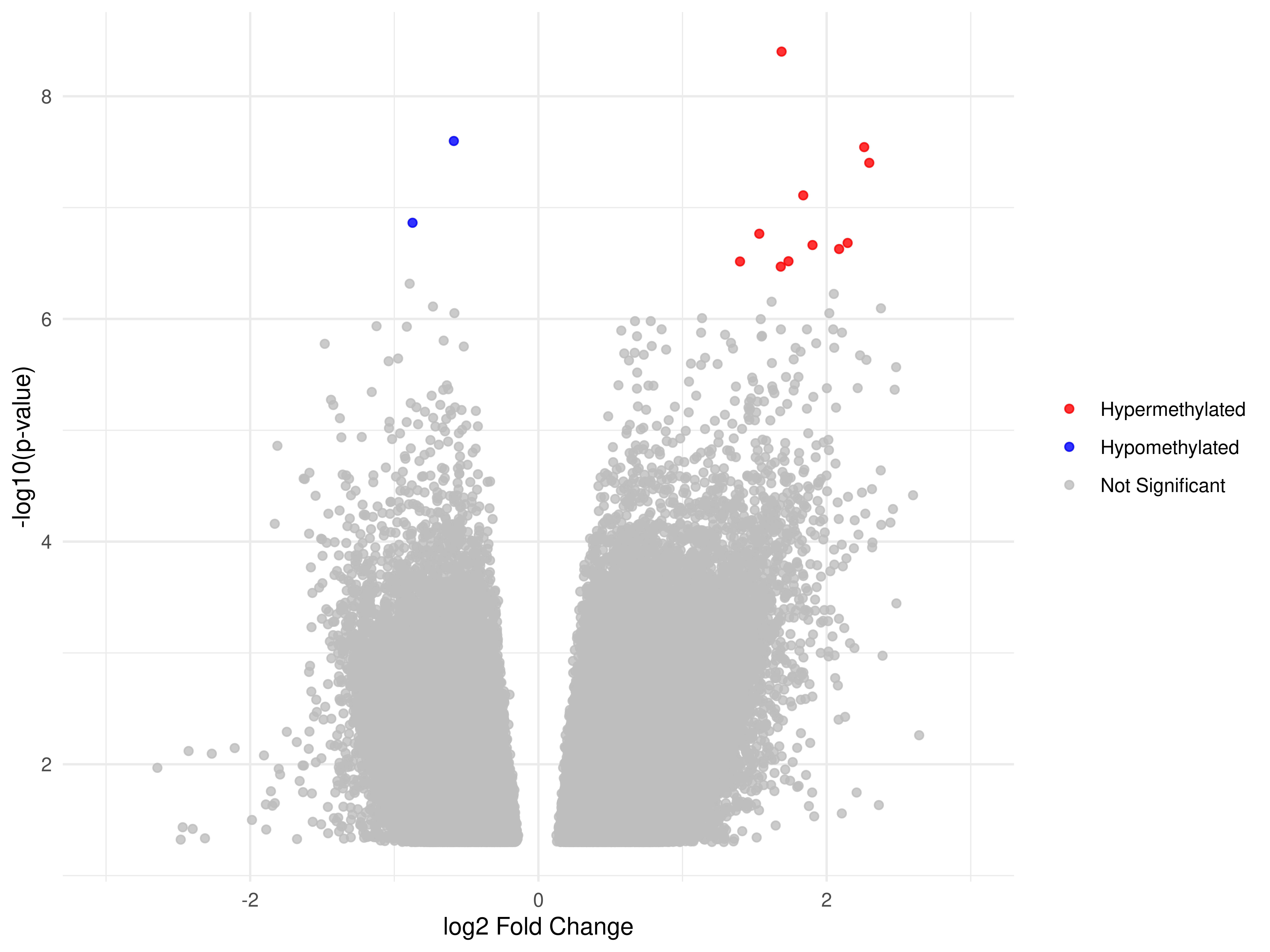

Supplement: Supplementary file 1 [file cancers-17-02026-s001.zip › Figure S8 - Volcano plot of DMRs between OC and healthy.png]
